# The prevalence and related factors of familial hypercholesterolemia in rural population of China using Chinese modified Dutch Lipid Clinic Network definition

**DOI:** 10.1186/s12889-019-7212-4

**Published:** 2019-06-27

**Authors:** Yan Wang, Yuqian Li, Xiaotian Liu, Runqi Tu, Haiqing Zhang, Xinling Qian, Jingjing Jiang, Dou Qiao, Xue Liu, Zhicheng Luo, Xiaokang Dong, Chongjian Wang

**Affiliations:** 1 Department of Epidemiology and Biostatistics, College of Public Health, Zhengzhou University, 100 Kexue Avenue, Zhengzhou, 450001 Henan People’s Republic of China; 2 Department of Clinical Pharmacology, School of Pharmaceutical Science, Zhengzhou University, Zhengzhou, Henan People’s Republic of China

**Keywords:** Familial hypercholesterolemia, Prevalence, Related factors, Rural population

## Abstract

**Background:**

Familial hypercholesterolemia (FH) is a common monogenic disease, while studies about the epidemiology for the general population in China was scarce. Aim of the study was to estimate the prevalence of FH and explore related factors by the Chinese modified Dutch Lipid Clinic Network (DLCN) definition.

**Methods:**

A total of 39,205 participants (15,463 males and 23,742 females) aged 18–79 years old were enrolled from the Henan Rural Cohort Study. FH was defined by the Chinese modified DLCN definition, and score > 5 was classified as FH. Logistic regression analysis was used to calculate the odds ratio and 95% confidence interval.

**Results:**

Crude prevalence of probable/definite FH was 0.35% (0.29–0.41%), estimated by the Chinese modified DLCN definition. Prevalence in female was 0.38%, and in male, it was 0.32%. Age-standardized prevalence in female increased significantly around the age of 50 years. Moreover, there were no FH patients getting low-density lipoprotein cholesterol controlled to the recommended level. Multivariate logistic regression identified that older, overweight/obesity were positively associated with FH.

**Conclusions:**

The current study indicated that FH was not rare in rural area of China (1 in 286). Effective early detection and timely control of FH must be strengthened to reduce disease burden.

**Electronic supplementary material:**

The online version of this article (10.1186/s12889-019-7212-4) contains supplementary material, which is available to authorized users.

## Background

Familial hypercholesterolemia is a relatively common autosomal monogenic disorder of lipid metabolism and associated with the dramatically increased lifetime risk of premature coronary artery disease due to the accelerated atherosclerosis [1]. Because of ischemic events from FH, life expectancy could be significantly shortened, especially the homozygous FH, which is characterized by the onset of premature cardiovascular disease in early childhood [2].

Several studies about FH have been conducted, while the prevalence among populations varies widely. Prevalence in the United States was reported to be 1 in 310 with low awareness and control [3]. While another study from the United States in the National Health and Nutrition Examination Survey showed a higher prevalence of 1 in 250 [4]. A DNA-analysis based study in a Japanese population identified the incidences of homozygous FH and heterozygous FH were 1/171,167 and 1/208, respectively [5]. Prevalence of Danish in Copenhagen General Population Study comprising 69,016 participants was estimated to be 0.73% (1 in 137) [6]. There were many diagnostic methods for FH, in which the Dutch Lipid Clinic Network (DLCN) has been widely used [7].

FH fulfills the World Health Organization criteria for population-based disease screening for early detection and treatment [8]. However, FH continues to be underestimated and undertreated in many areas [9]. Especially in China, the largest developing country, one study was conducted to estimate the prevalence of FH with Chinese modified DLCN in the general population, and the prevalence was shown to be 0.28% [10]. While other studies pertaining to FH in China, mainly included participants who were patients with myocardial infarction or undergoing coronary angiography [11]. Studies have shown that large-scale epidemiological investigations in China about FH are still scarce. There exist gaps between China and international community about Registry system and national management of FH [12, 13]. This phenomenon motivates studies to explore the prevalence in large-scale unselected population in more Chinese regions. Understanding what mainly are associated with FH will enable the development of strategies to prevent premature death and improve quality of life. Thus, the study aimed to investigate the epidemiology and related factors of FH in the rural areas in Henan, China. The Chinese modified DLCN diagnostic criteria [10] was used in this study.

## Methods

### Study participants

The Henan Rural Cohort, which was established during 2015–2017, is a large population-based study evaluating the prevalence and incidence of cardiometabolic disease and assessing health risk among Chinese rural population. The detailed information of our cohort has been previously published [14]. In brief, 39,259 rural participants completed face to face questionnaires. The age of participants ranged from 18 to 79 years old. And they come from 5 rural areas of Henan province including Xinxiang county, Tongxu county, Yuzhou county, Suiping county and Yima county. Fifty four subjects without information about low-density lipoprotein cholesterol (LDL-C) were excluded and the remaining 39,205 were included in the final analysis. This study was in line accordance with 1975 Declaration of Helsinki and approved by the Zhengzhou University Life Science Ethics Committee (Code: [2015] MEC (S128)). The written informed consent was obtained from every participant.

### Data collection

Detailed information of demographic variables, lifestyles, the history of disease and medication, family history of disease, and the presence of emotion and stress condition were collected as the baseline characteristics (Additional file 1). The demographic characteristics included sex, age, educational level (elementary school or below, junior high school and high school or above), income level (< 500, 500~, and ≥ 1000 renminbi (RMB)) and marital status (married/cohabitating and unmarried/divorced/widowed). Body mass index (BMI) was computed as individual weight (kg) divided by the height square (m^2^). According to the criteria recommended by Working Group on Obesity in China, BMI was grouped into two categories: low weight/normal weight (< 24.0 kg/m^2^) and overweight/obesity (≥ 24.0 kg/m^2^). Lifestyle behaviors included smoking (smoking at least one cigarette per day for sequential or cumulative half a year), alcohol consumption (drinking alcohol at least 12 times/year), diet habits, physical activity. High-fat diet was considered as the meat of livestock and poultry consumed by individual was more than 75 g per day. On the basis of International Physical Activity Questionnaire [15], physical activity was grouped into three categories including low, moderate and high level. More vegetable and fruit intake was considered as the vegetable and fruit consumed by individual beyond 500 g/day. After at least eight hours of fasting, the venous blood samples were gathered from individuals and separated through centrifugation. Roche Cobas C501 automatic biochemical analyzer was used to analyze total cholesterol (TC), triglyceride (TG), high-density lipoprotein cholesterol (HDL-C) and LDL-C. Direct method was taken to estimate HDL-C and LDL-C. Cholesterol oxidase method was used to analyze TC, while enzymatic method was taken to estimate TG.

### Definition of FH

FH was defined according to the Chinese modified DLCN criteria [10] which included family history, personal history and the levels of LDL-C. The details and corresponding point are as follows:

- Family history of a first-degree relative with known premature coronary artery disease or vascular disease (the age was younger than 60 years old when the first-degree relative was diagnosed, 1 point);
- Personal history of premature coronary artery disease (male was under 55 years old when he was diagnosed; female was under 60 years when she was diagnosed, 2 points) or premature cerebral vascular disease (male was under 55 years old when he was diagnosed; female was under 60 years when she was diagnosed, 1 point);
- LDL-C ≥ 6 mmol/L (8 points), 5.0–5.9 mmol/L (5 points), 3.5–4.9 mmol/L (3 points) and 2.5–3.4 mmol/L (1 point).
- FH was classified as four categories according to total score (> 8: definite; 6–8: probable; 3–5: possible; < 3: unlikely). Phenotypic FH was defined as score > 5.

### Statistical analysis

The student’s t-test and chi-squared test were respectively used to compare continuous variables and categorical variables among participants. Furthermore, continuous variables were presented as mean (plus and minus standard deviation), and categorical variables were presented as numbers and proportions. The odds ratio (OR) and 95% confidence interval (95%CI) between demographic or anthropometric characteristics and FH were calculated by logistic regression model. On the basis of the sixth Population Census, prevalence of FH was standardized using the direct method. SAS 9.1 software package (SAS Institute, USA) was performed to conduct the statistical analyses. The statistical significance level was set at α = 0.05.

## Results

### Characteristic of participants

In total, 39,205 subjects were included, among which female account for 60.56%. According to the Chinese modified DLCN criteria, compared with unlikely/possible FH participants, probable/definite FH participants were more likely to be older ages and higher BMI. No significant difference between other characteristics was observed in different FH categories (Table 1).

**Table 1:** The characteristic of the participants

Variables	Chinese Modified DLCN (*N* = 39,205)		
	Unlikely/possible	Probable/definite	*P*-value
Number	31,044/8022	138/1	
Age, mean (SD)			0.019
< 60	22,115 (56.61)	65 (46.76)	
≥ 60	16,951 (43.39)	74 (53.23)	
Gender			0.311
Male	15,414 (39.46)	49 (35.25)	
Female	23,652 (60.54)	90 (64.75)	
Marital status, n (%)			0.826
Married/cohabiting	35,071 (89.77)	124 (89.21)	
Unmarried/divorced/widowed	3995 (10.23)	15 (10.79)	
Education, n (%)			0.079
Elementary school or below	17,478 (44.74)	75 (53.95)	
Junior high school	15,577 (39.87)	44 (31.65)	
High school or above	6011 (15.39)	20 (14.39)	
Average monthly individual income, n (%)			0.230
< 500RMB	13,937 (35.67)	51 (36.69)	
500~RMB	12,859 (32.92)	37 (26.62)	
1000~RMB	12,270 (31.41)	51 (36.69)	
Smoking, n (%)			0.744
No smoking	31,614 (80.92)	114 (82.01)	
Current smoking	7452 (19.08)	25 (17.99)	
Drinking, n (%)			0.180
No drinking	32,016 (81.95)	120 (86.33)	
Current drinking	7050 (18.05)	19 (13.67)	
Physical activity, n (%)			0.121
Low	12,634 (32.34)	56 (40.29)	
Moderate	14,746 (37.75)	44 (31.65)	
High	11,686 (29.91)	39 (28.06)	
High-fat diet, n (%)			0.747
No	31,619 (80.94)	114 (82.01)	
Yes	7447 (19.06)	25 (17.99)	
More vegetable and fruit intake, n (%)			0.225
No	22,746 (58.23)	88 (63.31)	
Yes	16,318 (41.77)	51 (36.69)	
BMI, mean (SD)	24.83 (3.57)	25.46 (3.12)	0.020

*SD* standard deviation, *RMB* renminbi, *BMI* body mass index

### Prevalence and treatment of FH

In this study, according to Chinese modified DLCN, overall crude and age-standardized prevalence of FH were 0.35% (0.29–0.41%) and 0.30% (0.25–0.35%). Crude prevalence in female was 0.38% (0.30–0.46%), in male it was 0.32% (0.23–0.41%). Age-standardized prevalence in female was 0.25% (0.19–0.31%) and in male it was 0.38% (0.28–0.48%). Participants who were older and overweight/obese had a significantly higher prevalence of FH (Table 2). Furthermore, among the 139 definite/probable FH patients, 13 (9.35%) were receiving medication treatment.

**Table 2:** Prevalence and 95% confidence interval of FH among participants according to definition

Variable	Chinese Modified DLCN (*n* = 39,205)	
	Probable/definite	*P*-value
Number	138/1	
Age (years), n (%)		0.019
< 60	0.29 (0.22–0.36)	
≥ 60	0.43 (0.34–0.53)	
Gender		0.311
Male	0.32 (0.23–0.41)	
Female	0.38 (0.30–0.46)	
Marital status, n (%)		0.826
Married/cohabiting	0.35 (0.29–0.41)	
Unmarried/divorced/widowed	0.37 (0.19–0.56)	
Education, n (%)		0.079
Elementary school or below	0.43 (0.33–0.52)	
Junior high school	0.28 (0.20–0.36)	
High school or above	0.33 (0.19–0.48)	
Average monthly individual income, n (%)		0.230
< 500RMB	0.36 (0.26–0.46)	
500~RMB	0.29 (0.19–0.38)	
1000~RMB	0.41 (0.30–0.53)	
Smoking, n (%)		0.744
No smoking	0.36 (0.29–0.43)	
Current smoking	0.33 (0.20–0.47)	
Drinking, n (%)		0.180
No drinking	0.37 (0.31–0.44)	
Current drinking	0.27 (0.15–0.39)	
Physical activity, n (%)		0.121
Low	0.44 (0.33–0.56)	
Moderate	0.30 (0.21–0.39)	
High	0.33 (0.23–0.44)	
High-fat diet, n (%)		0.747
No	0.36 (0.29–0.43)	
Yes	0.33 (0.20–0.47)	
More vegetables and fruit intake, n (%)		0.225
No	0.39 (0.31–0.47)	
Yes	0.31 (0.23–0.40)	
BMI, kg/m^2^		0.001
Low weight/normal weight	0.24 (0.17–0.31)	
Overweight/obesity	0.44 (0.35–0.52)	

*RMB* renminbi, *BMI* body mass index

**Fig. 1:**
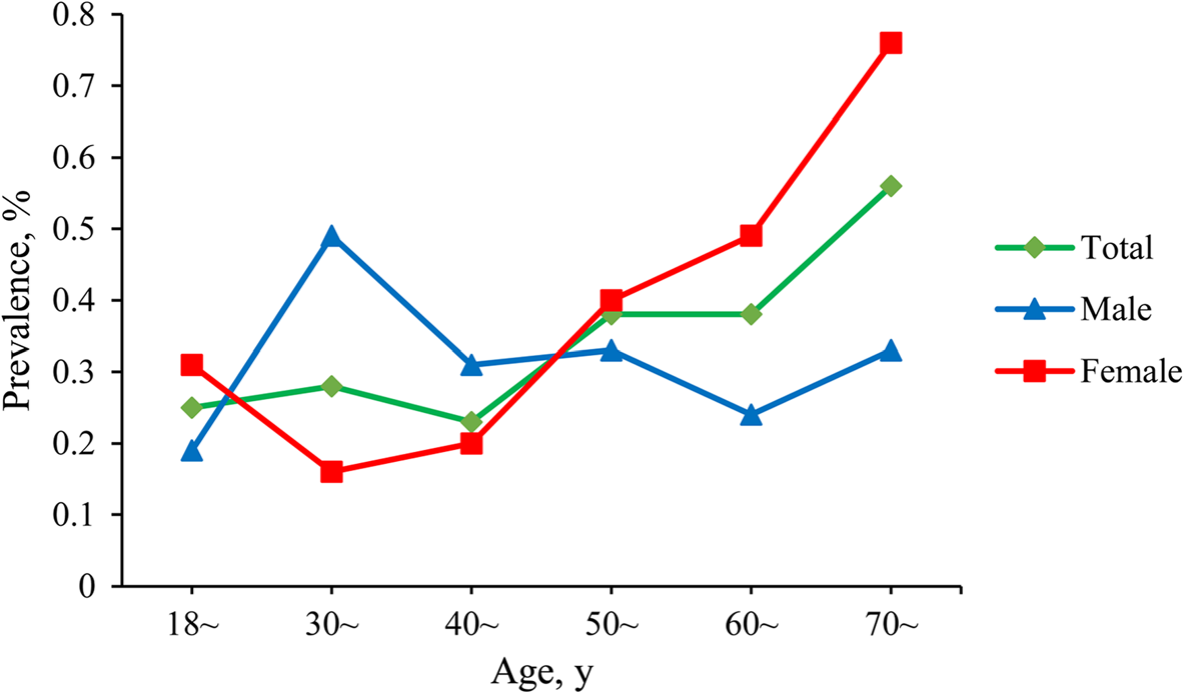
The age-standardized prevalence of FH among different age groups using Chinese modified DLCN definition

Figure 1 shows the age-standardized prevalence for total subjects, male and female among different age groups based on the Chinese modified DLCN definition. Males in the 30~ age group showed the highest age-standardized prevalence. For females, the age-standardized prevalence showed a marked increase around the age of 50 years according to the Chinese modified definition.

### Lipid levels

Mean levels of TC, TG, HDL-C and LDL-C were 4.75, 1.68, 1.32 and 2.87 mmol/L respectively. The mean levels of all lipids in female was significantly higher than male (Table 3).

**Table 3:** The mean level of lipid among genders

Lipid	Total	Male	Female	*P*-value
TC (mmol/L)	4.75 (4.74–4.76)	4.63 (4.62–4.65)	4.83 (4.82–4.84)	< 0.001
TG (mmol/L)	1.68 (1.66–1.69)	1.66 (1.64–1.68)	1.69 (1.67–1.70)	0.027
HDL-C (mmol/L)	1.32 (1.31–1.33)	1.26 (1.25–1.27)	1.36 (1.35–1.37)	< 0.001
LDL-C (mmol/L)	2.87 (2.86–2.88)	2.83 (2.82–2.84)	2.90 (2.89–2.91)	< 0.001

Table 4 shows the concentration of plasma lipid among FH diagnostic groups with or without cholesterol-lowering medication. According to the Chinese modified definition, individuals defined as definite/probable FH not treated with cholesterol-lowering medication showed 151% higher LDL-C level (6.44 vs. 2.57) compared with those who were unlikely to suffer FH and not treated with cholesterol-lowering medication. Definite/probable FH subjects treated with cholesterol-lowering medication had 193% higher LDL-C level (7.18 vs.2.45) compared with who were unlikely to suffer FH and treated with cholesterol-lowering medication.

**Table 4:** Lipid level among participants with FH on and off cholesterol-lowering medication

	FH category					
	Definite/probable		Possible		Unlikely	
	Off	On	Off	On	Off	On
Chinese modified DLCN, n	126	13	7551	471	29,673	1371
TC (mmol/L)	8.21 (1.11)	9.09 (1.73)	5.91 (0.75)	5.93 (0.80)	4.45 (0.76)	4.45 (0.89)
TG (mmol/L)	2.02 (1.09)	2.65 (1.59)	1.76 (0.87)^a^	2.02 (0.99)	1.62 (1.15)^a^	2.21 (1.51)
HDL-C (mmol/L)	1.42 (0.33)	1.45 (0.35)	1.38 (0.32)^a^	1.34 (0.30)	1.31 (0.34)^a^	1.22 (0.33)
LDL-C (mmol/L)	6.44 (0.70)	7.18 (2.01)	3.99 (0.52)	3.97 (0.60)	2.57 (0.55)^a^	2.45 (0.64)

^a^*P* < 0.05 for the difference between participants off and on cholesterol-lowering medication among FH categories

### Logistic regression analysis

The results of multivariate logistic regression in the Chinese modified DLCN showed that older age and overweight/obesity were positively associated with FH, while no significant association was found among other characteristics (Table 5).

**Table 5:** Odds ratio (95% CI) of FH among different characteristics

Variables	Chinese Modified DLCN	*P-*value
Age	1.02 (1.01–1.04)	0.011
Gender		
Male	1.00	
Female	1.24 (0.76–2.01)	0.391
Marital status, n (%)		
Married/cohabiting	1.00	
Unmarried/divorced/widowed	0.90 (0.52–1.56)	0.697
Education, n (%)		
Elementary school or below	1.00	
Junior high school	0.82 (0.54–1.24)	0.346
High school or above	1.02 (0.58–1.77)	0.955
Average monthly individual income, n (%)		
< 500RMB	1.00	
500~RMB	0.86 (0.56–1.32)	0.488
1000~RMB	1.32 (0.88–1.99)	0.185
Smoking, n (%)		
No smoking	1.00	
Current smoking	1.30 (0.74–2.29)	0.361
Drinking, n (%)		
No drinking	1.00	
Current drinking	0.76 (0.43–1.33)	0.335
Physical activity, n (%)		
Low	1.00	
Moderate	0.72 (0.48–1.08)	0.117
High	0.83 (0.55–1.26)	0.389
High-fat diet, n (%)		
No	1.00	
Yes	1.09 (0.69–1.72)	0.706
More vegetables and fruit intake, n (%)		
No	1.00	
Yes	0.86 (0.61–1.23)	0.417
BMI		
Low weight/normal weight	1.00	
Overweight/obesity	1.86 (1.28–2.69)	0.001

*RMB* renminbi, *BMI* body mass index. All variables were adjusted in the multivariable model and age was treated as a continuous variable

## Discussion

The present study provided up-to-date prevalence of FH and the related factors based on the unselected Chinese rural population aged 18–79 years. According to Chinese modified DLCN, the overall crude prevalence of FH was 0.35% (0.29–0.41%), and the age-standardized prevalence of total population was 0.30% (0.25–0.35%). The crude prevalence of FH was 0.38% in females and 0.32% in males. The estimate in our study was comparable with previous meta-analysis report of 0.46% (0.25–0.70%) based on DLCN [16]. A previous study in 2007 conducted in China suggested that overall crude prevalence of FH in Jiangsu was 0.28% using modified DLCN definition and 0.47% using LDL based definition [10]. The present study indicated that the crude prevalence of rural population of Henan was similar to the study above.

In the present study, the mean levels of TC, TG and LDL-C were higher, while HDL-C levels were lower compared with the national level of rural adult Chinese [17]. In the current study, in the Chinese modified DLCN criteria, the proportion of patients who were treated was 9.35%. Furthermore, it is the same as the Jiangsu study, that although some patients were treated with cholesterol-lowering medication, there was none that met the LDL-C target (< 2.5 mmol/L) [7]. Moreover, previous studies in European populations reported that a significant proportion of subjects at risk of coronary artery disease did not meet the recommended plasma level of LDL-C, even if they were on treatment with statin [18–20]. Thus, more effective primary prevention should be taken for keeping people healthy and decreasing the incidence of cardiovascular events for the general population.

A previous study suggested that the estimated prevalence of FH in younger participants was lower than in older subjects and this phenomenon might be attributable to lower prevalence and fewer self-reported family history of coronary artery disease, which implied that FH remains under-detected [6]. The present study also demonstrated that the prevalence of FH in older participants was higher than younger participants. Moreover, a review of FH has illustrated that age was linked with the increased risk of coronary artery disease among Chinese FH patients [12]. Importantly, early and timely detection of FH among children and young adults for the prevention of premature cardiovascular disease was urgently needed.

A previous study found that postmenopausal and late menopausal transition period were both in correlation with the lipid and lipoprotein abnormalities [21]. Besides, a previous research from the USA found differences in FH prevalence among different age groups and obesity status, in addition, the study suggested that the effect of age or obesity on LDL-C level might be a result of enhanced penetrance with changing environmental factors, or LDL-C level showed a physiological increase because of age or menopause [4]. In the present study, we also found that the prevalence of FH increased around 50 years old in female, and the differences in FH prevalence were also seen in different age groups and obesity status. More related researches about FH in genetic testing should be conducted in the future.

The present study analyzed the epidemiology and associated factors of FH based on a relatively large sample in Chinese rural area. Besides, trained staff, standardized survey tools, precise equipment and adjusting for a large range of covariables ensured the reliability of the research. However, there existed some limitations. Firstly, modified DLCN criteria was used in our study, not including genetic testing. Nevertheless, the modified DLCN was more appropriate in large-scale general population and field implementation. Secondly, only 4.73% (1855/39205) of the participants reported treatment with cholesterol-lowering medication, and we estimated the prevalence without adjustment for lipid-lowering medication. What we should pay attention to was that correction factors had inherent pitfalls of both overestimation and underestimation of the true level of LDL-C in participants with treatment [4]. Thirdly, representation of the current study was limited because of the geographical region design.

## Conclusions

Prevalence of FH was 1 in 286 when using the Chinese modified DLCN. None was treated to recommended level in individuals with lipid-lowering treatment. Further research should be carried out for screening potential FH. Health education and preventive strategies of FH should be conducted to reduce the disease burden.

## Additional file

Additional file 1: **Table S1.** The English version of questionnaire. (PDF 624 kb)

## Data Availability

The data used in this study are available and will be provided by the corresponding author on a reasonable request.

## Abbreviations

BMI: Body mass index
CI: Confidence interval
DLCN: Dutch lipid clinic network
FH: Familial hypercholesterolemia
HDL-C: High-density lipoprotein cholesterol
LDL-C: Low-density lipoprotein cholesterol
OR: Odds ratio
SD: Standard deviation
TC: Total cholesterol
TG: Triglyceride
