# Supplementary material for: The prevalence and related factors of familial hypercholesterolemia in rural population of China using Chinese modified Dutch Lipid Clinic Network definition
Source: BMC Public Health. 2019 Jun 27;19:837. doi: 10.1186/s12889-019-7212-4 (PMC6598239; doi:10.1186/s12889-019-7212-4)
Supplement: Supplementary file 1 — Table S1. The English version of questionnaire. (PDF 624 kb) [file 12889_2019_7212_MOESM1_ESM.pdf]

Name： ___________ **Study ID:□□□□□□**

Common chronic diseases and health influencing factors in rural residents

College of Public Health, Zhengzhou university

Zhengzhou, Henan province

Informed Consent

Along with the rapid growth of social economy, improvement of people's living standards, changes in diet and aging of population, morbidity and mortality of the common chronic diseases such as hypertension, diabetes are rapidly rising. The trend is also more and more obvious in younger. Thus, social, family and personal economic burden is getting heavier and heavier. We invite you to participate in the physical examination of common chronic diseases, including questionnaire survey and physical examination. The whole process can be completed in 1-2 hours.

Objective: physical examination is an important measure for early detection, early diagnosis and early treatment of diseases. It can not only help you get precious time for timely treatment of diseases, but also reduce your physical and mental suffering and unnecessary financial burden. Through health examination, patients with common chronic diseases and high-risk groups can be screened, which can promote the education and prevention of chronic diseases and reduce the risk of chronic diseases.

Main research institution: College of public health, Zhengzhou university.

Contact person on site: Person in charge or doctor on site.

Screening criteria: Health survey and physical examination were conducted on family members aged 18-79 years.

Benefits: Physical examination, blood pressure measurement, blood and urine test can identify the risk factors and high-risk groups of common chronic diseases such as hypertension, diabetes, obesity, hyperlipidemia, coronary heart disease and stroke as early as possible. After the physical examination, you will be provided with the results of blood routine, urine routine, electrocardiogram, pulmonary function, abdominal B ultrasound, chest X-ray (except for pregnant and lying-in women), blood lipid, liver function and kidney function.

Possible risk: You may have slight pain when drawing blood.

Selectivity and rights: The physical examination will ask for your permission. If you take part in the physical examination, the doctor can help you measure blood pressure, body measurement, blood routine, urine routine, electrocardiogram, pulmonary function, abdominal B ultrasound, chest X-ray, blood lipid, liver and kidney function test, etc. At the same time, the knowledge of prevention and treatment of common chronic diseases is publicized. You may decide to decline or withdraw from the study and it does not affect your normal medical services.

Privacy protection: The investigation results will be submitted to the college of

public health of Zhengzhou university. The scientific results of the research will be published, but any personal privacy information will not be disclosed to others. We will protect your personal information.

Examination fee: The above investigation and examination are free of charge. College of public health of Zhengzhou university and physical examination institutions provide funds for you to carry out the relevant health examination.

Signature: I have read the informed consent and I am willing to participate in this project.

Participant signature： ______________ Date： ____________

Witness signature： ______________ Date： ____________

The questionnaire of common chronic diseases and health influencing factors in rural residents

**1.** Householder name: ______________	**2.** Name: ______________
**3.** ID number: □□□□□□□□□□□□□□□□□□	
**4.** Home address: ____City____District/County____Town_____Village/Street____Team____Number	
**5.** Telephone: _________________(Home)____________________(Cellphone)	
**6.** Contact name: _________Relation: ________Telephone (different from above): _________________	
**7.** Study ID: □□□□□□	**8.** ID of survey site: □□
**9.** Investigation date: ______Year____Month____Day	
**10.** Time of study onset: _____h____ mins	

1. General characteristics

**A1.** Residence: 1=Urban 2=Rural □	**A2.** Gender: 1=Male 2=Female □
**A3.** Nation: 1=Han 8=Other_______ □	**A4.** Religion: 0=No 1=Have______ □
**A5.** Birthdate: _____Year___Month___Day 1=Solar calendar 2=Lunar calendar □	
**A6.** Education level: □<br>1=No formal school 4=High School/Technical school<br>2=Primary School 5=Junior college/University<br>3=Middle School 6=Master or above	
**A7.** Marital status: □<br>1=Married/cohabitation 2=Widowed 3=Separated/divorced 4=Unmarried	
**A8.** Occupation: □<br>1=Worker (including migrant worker) 6=Self-employed	

2=Farmer 7=Retired

3=Administrator/cadre 8=House wife/husband

4=Professional/technical 9=Other (indicate： __________)

5=Sale & service workers

A9. Number of family population:__________

Total income last year in your household:__________yuan?

A10. What is the level of your per capita monthly income of families (yuan)? ☐

1= <500 2= 500~999 3= 1000~1999 4= 2000~2999 5= ≥3000

A11. Do you have any medical service? (please choose the main one) ☐

1=New rural cooperative medical system 4=Commercial medical insurance

2=Medical insurance for urban employees 5=Free medical service

3= Medical insurance for urban residents 8=Other__________

A12. What kind of hospital do you often go to? (please choose the main one) ☐

1=Village clinic

2=Township hospital/community medical unit

3=County and district hospital/second-class hospital

4=Municipal hospital/first-class hospital

5=Private hospital

9=Unknown

2. Lifestyle

(2.1) Smoking history

B1. Do you smoke? (smoking at least one cigarette a day for more than six months). ☐

0=Never or only occasionally (not more than one cigarette per day on average） (*Go to **B2***)

1=Current smoking	**B1a.** Start smoking： _____years old<br>**B1b.** Average number of smoking per day： _______ cigarette (*Go to **B3***)

2=Quit smoking	**B1c.** Start smoking： _____years old<br><br>**B1d.** Average number of smoking per day before quit： _____cigarette<br><br>**B1e.** Age at the time of smoking cessation： _____years old<br><br>**B1f.** Your main reason for stopping： □<br><br>1=Money 2= Health concerns<br><br>3=Family/friend against 8=Other (indicate： _________) (*Go to **B3***)
**B2.** Have you ever inhaled smoke from other people`s cigarette (passive smoking：Non-smokers inhale smoke from smokers for at least 15 minutes a day)?	
0=Never or basically no (*Go to **B3***)	
1=Yes	**B2a.** Passive smoking per week:_______days （9=unknown）
	**B2b.** Passive smoking per day:______hour______minutes （99=unknown）
	**B2c.** Duration of passive smoking:________year
**(2.2) Alcohol consumption**	
**B3.** Do you drink (drinking at least 12 times a year)? □	
0=Never or only occasionally (*Go to **B7***)	
1=Current drinking	**B3a**. Start drinking： _______years old (*Go to **B4***)
2= Quit drinking	**B3b.** Start drinking： _______years old<br><br>**B3c.** Age at the time of stopping drinking： _____years old<br><br>**B3d.** Your main reason for stopping： □<br><br>1=Money 2= Health concerns<br><br>3=Family/friend against 8=Other (indicate： _________)
**B4.** On days when you drink, how often do you drink alcohol? (including current or quit drinking) □	

1=At least 1 time/day 2=At least 1 time/week 3=At least 1 time/month
**B5.** Type, frequency and amount of alcohol consumed (including current or quit drinking)

Alcohol type	Frequency (select one)	Times	Amount/times	Month/year
**B5a.** Beer	0=never 1=day 2=week 3=month 4=year	_____	____bottle	_________
**B5b.** Liquor	0=never 1=day 2=week 3=month 4=year	_____	____liang	_________
**B5c.** Red wine	0=never 1=day 2=week 3=month 4=year	_____	____liang	_________
**B5d.** Rice wine	0=never 1=day 2=week 3=month 4=year	_____	____liang	_________

B6. Are you often drunk? (including current or quit drinking) ☐

1=Almost every time 2=Vast majority 3=Occasionally 4=Never

(2.3) Diet

B7. During the past 12 months, do you cook at home (at least one time per week)? ☐

0=No (*Go to **B12***) 1=Yes

B8. You cook at home____times/week, the duration you cook every time on average was____minutes.

B9. When you cook at home, which type of cooking oil do you mainly use (select one)? ☐

1=Soybean oil 2=Peanut oil 3=Animal oil 4= Rapeseed oil 8=Other_________ 9=Unknown

B10. When you cook at home, which type of stove do you mainly use (select one)? ☐

1=Firewood stove 2=Induction cooker 3=Coal gas 4=Coal stove 5=Natural gas 8=Other________

B11. When you cook at home, what is the main ventilation method in your kitchen (select one)? ☐

1=Range hood 2=Exhaust fan 3=Window ventilation 8=Other_________

B12. During the past week, you had breakfast_______times/week, you ate out______times/week.

B13. During the past week, you had lunch_______times/week, you ate out______times/week.

B14. During the past week, you had dinner_______times/week, you ate out______times/week.

Food intake (<u>Record the frequency and amount of food you ate in the past year</u>)

Type	Frequency (select one)	Amount
**B15.** Staple food (rice and wheat)	0=never 1=day 2=week 3=month 4=year ☐	___catty____liang
**B16.** Pork, beef and mutton	0=never 1=day 2=week 3=month 4=year ☐	___catty____liang
**B17.** Chicken and duck	0=never 1=day 2=week 3=month 4=year ☐	___catty____liang
**B18.** Fish	0=never 1=day 2=week 3=month 4=year ☐	___catty____liang
**B19.** Eggs, duck eggs, etc.	0=never 1=day 2=week 3=month 4=year ☐	___(number)
**B20.** Dairy products (milk, yogurt)	0=never 1=day 2=week 3=month 4=year ☐	____ml
**B21.** Fresh fruits	0=never 1=day 2=week 3=month 4=year ☐	___catty____liang
**B22.** Fresh vegetables	0=never 1=day 2=week 3=month 4=year ☐	___catty____liang
**B23.** Soybean products	0=never 1=day 2=week 3=month 4=year ☐	___catty____liang
**B24.** Nuts (melon seed, peanut, etc.)	0=never 1=day 2=week 3=month 4=year ☐	___catty____liang
**B25.** Pickles	0=never 1=day 2=week 3=month 4=year ☐	___catty____liang
**B26.** Other staple food (corn, potato, sorghum, etc.)	0=never 1=day 2=week 3=month 4=year ☐	___catty____liang
**B27.** Animal oil (lard/butter)	0=never 1=day 2=week 3=month 4=year ☐	___catty____liang

B28. Your taste

B28a. Strength of salty food you prefer to eat： 1=Mild 2=Middle 3= Heavy 4= Extremely heavy ☐

B28b. Strength of sweet food you prefer to eat： 1=No 2=Mild 3= Middle 4=Heavy ☐

B28c. Strength of sour food you prefer to eat： 1=No 2=Mild 3= Middle 4=Heavy ☐

B28d. Strength of spicy food you prefer to eat： 1=No 2=Mild 3= Middle 4=Heavy ☐

B29. During the past month, do you eat spicy food (mainly refers to chili)? ☐

0=No (*Go to **B31***) 1=Yes, _______days/week

B30. During the past month, which kind of chili do you usually eat? ☐

1= Chili sauce 2= Chili oil 3= Dried chili 4= Fresh pepper 8=Other__________ 9=Unknown

B31. Do you often drink tea? (usually refers to drinking tea at least once a week for more than 6 months) ☐

0=No (*Go to **C1***) 1=Yes

B32. What kind of tea do you usually drink? ☐

1= Green tea 2= Black tea

3= Scented tea 8=Other________

B33. You drink the tea_________days per week.

(2.4) Physical Activity (The questions will ask you about the time you spent on physical activity in the last 7 days.)

Think about all the vigorous activities that you did in the last 7 days. Vigorous physical activities refer to activities that take hard physical effort and make you breathe much harder than normal. Think only about those physical activities that you did for at least 10 minutes at a time.

C1. During the last 7 days, on how many days did you do vigorous physical activities like heavy lifting, digging, aerobics, or fast bicycling? ☐

0=No vigorous physical activity (*Go to **C3***) 1=________days per week?

C2. How much time did you usually spend doing vigorous physical activities on one of those days? ☐

0=Don't know/Not sure. 1=_______hours per day?_____minutes per day?

Think about all the moderate activities that you did in the last 7 days. Moderate physical activities refer to activities that take moderate physical effort and make you somewhat harder than normal. Think only about those physical activities that you did for at least 10 minutes at a time.

C3. During the last 7 days, on how many days did you do moderate physical activities like carrying light loads, bicycling at a regular pace, or doubles tennis? Please do not include walking. ☐

0=No moderate physical activity (*Go to **C5***) 1=_______days per week?

C4. How much time did you usually spend on one of those days doing moderate physical activities as part of your life? ☐

0=Don’t know/Not sure.   1=_______hours per day?_____minutes per day?

Think about all time you spent walking in the last 7 days. This includes at work and at home, walking to travel from place to place, and any other walking that you might do solely for recreation, sport, exercise, or leisure.

C5. During the last 7 days, on how many days did you walk for at least 10 minutes at a time? ☐

0= No walking (*Go to* ***C7***)   1=______days per week?

C6. How much time did you usually spend walking on one of those days? ☐

0= Don’t know/Not sure.   1=_______hours per day?_____minutes per day?

The last question is about the time you spent sitting on weekdays during the last 7 days. This include time spent at work, at home, while doing course work and during leisure time. This may include time spent sitting at a desk, visiting friends, reading, or sitting or lying down to watch television.

C7. During the last 7 days, how much time did you spend sitting? ☐

0=Don’t know/Not sure.   1=_______hours per day?_____minutes per day? Of which, sitting to watch television /DVD/computer/mobile phone_______hours per day?_____minutes per day?

(2.5) Sleep Condition

D1. Do you often take a nap in the last six months? ☐

0=No (*Go to* ***D3***)   1=Yes, _____days/week,_______hours per day?_____minutes per day?

D2. How do you feel the quality of your nap? ☐

1=Very good   2=Better   3=Poor   4=Very bad

D3. Have you been working night shifts in the last six months? ☐

0=No   1=Yes (________days/month)

D4. Last month, in the night, you usually go to sleep at___hour___minutes.

The duration you need to fall asleep:___minutes

In the morning, you usually get up at_____hour_____minutes.   (*Please fill in 24h*)

**D5.** The following situation in your most recent month				
**Sleep situation**	**No**	**<1time/week**	**1~2times/week**	**≥3times/week**
**D5a.** Difficulty in falling asleep at night (refers to not falling asleep within 30 minutes)	1	2	3	4
**D5b.** Easy to wake up or wake up early	1	2	3	4
**D5c.** Get up at night to go to the toilet	1	2	3	4
**D5d.** Disturbance in respiration during sleep at night	1	2	3	4
**D5e.** Coughing or snoring during sleep at night	1	2	3	4
**D5f.** Feeling cold during sleep at night	1	2	3	4
**D5g.** Feeling too hot during sleep at night	1	2	3	4
**D5h.** Nightmare during sleep at night	1	2	3	4
**D5i.** Painful discomfort during sleep at night	1	2	3	4
**D5j.** Other conditions that affect nighttime sleep (if so, please indicate_________)	1	2	3	4
**D5k.** Need to take medication (including prescription from a doctor or at an outside pharmacy) to fall asleep?	1	2	3	4
**D5l.** It is difficult to stay awake when driving, eating or participating in social activities	1	2	3	4
**D5m.** How do you feel about the quality of your nighttime sleep?	Very good	Better	Poor	Very bad
**D5n.** Are you having trouble working on things actively?	No	Slight	difficult	Very difficult

3. History of disease, medication and familial disease (Fill in according to the doctor/hospital diagnosis result)

Disease name	Personal medical history (Age at diagnosis)	Drug type	Have you used the above drugs? (usage time)	Have you used these drugs in the last two weeks?	History of disease in immediate family (Multiple choice)					
					Father	Mother	Siblings	Children	Unknown	No
**E1.** Hypertension	0=No 1=_____years	Antihypertensive drugs	0=No 1=(____year____month)	0=No 1=Yes	□__years	□__years	□__years	□__years	□	□
**E2.** Hyperlipidemia	0=No 1=_____years	Lipid lowering drug	0=No 1=(_____year____month)	0=No 1=Yes	□__years	□__years	□__years	□__years	□	□
**E3.** Diabetes	0=No 1=_____years	Oral hypoglycemic agent	0=No 1=(_____year____month)	0=No 1=Yes	□__years	□__years	□__years	□__years	□	□
		Insulin	0=No 1=(_____year____month)	0=No 1=Yes						
**E4.** Coronary heart disease	0=No 1=_____years	Therapeutic drugs	0=No 1=(_____year____month)	0=No 1=Yes	□__years	□__years	□__years	□__years	□	□
**E4a.** If so	(Multiple choice)：1= Myocardial infarction 2= Angina pectoris 3= Arrhythmia 4= Heart failure 8=Other_______________ 9=Unknown □									
**E5.** Stroke	0=No 1=_____years	Therapeutic drugs	0=No 1=(_____year____month)	0=No 1=Yes	□__years	□__years	□__years	□__years	□	□
**E5a.** If so	(Multiple choice)：1= Ischemic stroke 2= Hemorrhagic stroke 8=Other_______________ 9=Unknown □									
**E6.** Emphysema	0=No 1=_____years	Therapeutic drugs	0=No 1=(_____year____month)	0=No 1=Yes	□__years	□__years	□__years	□__years	□	□

**E7.** Chronic bronchitis	0=No 1=_____years	Therapeutic drugs	0=No 1=(_____year____month)	0=No 1=Yes	□__years	□__years	□__years	□__years	□	□
**E8.** Asthma	0=No 1=_____years	Therapeutic drugs	0=No 1=(_____year____month)	0=No 1=Yes	□__years	□__years	□__years	□__years	□	□
**E9.** COPD	0=No 1=_____years	Therapeutic drugs	0=No 1=(_____year____month)	0=No 1=Yes	□__years	□__years	□__years	□__years	□	□
**E10.** Cancer	0=No 1=_____years	Antineoplastic drugs	0=No 1=(_____year____month)	0=No 1=Yes	□__years	□__years	□__years	□__years	□	□
**E10a.** If so	Please fill in the name of cancer：______________________, _____________________, _______________________									
**E11.** Kidney disease	0=No 1=_____years	Therapeutic drugs	0=No 1=(_____year____month)	0=No 1=Yes	□__years	□__years	□__years	□__years	□	□
**E11a.** If so	(Multiple choice)：1= Kidney stones 2= Nephritis 3= Renal cyst 4= Renal failure 5= Ureteral calculi 8=Other______________ 9=Unknown □									
**E12.** Liver Disease	0=No 1=_____years	Therapeutic drugs	0=No 1=(_____year____month)	0=No 1=Yes	□__years	□__years	□__years	□__years	□	□
**E12a.** If so	(Multiple choice)：1= Fatty liver 2= Cirrhosis 3= Hepatic cyst 4= Liver abscess 8=Other______________ 9=Unknown □									
**E13.** Chronic hepatitis	0=No 1=_____years	Therapeutic drugs	0=No 1=(_____year____month)	0=No 1=Yes	□__years	□__years	□__years	□__years	□	□
**E13a.** If so	(Multiple choice)：1= Viral hepatitis 2= Alcoholic hepatitis 3= Drug-induced hepatitis 8=Other______________ 9=Unknown □									
**E14.** Gallbladder disease	0=No 1=_____years	Therapeutic drugs	0=No 1=(_____year____month)	0=No 1=Yes	□__years	□__years	□__years	□__years	□	□

**E14a.** If so	(Multiple choice)：1= Gallstone 2= Cholecystitis 8=Other______________ 9=Unknown									□
**E15.** Tuberculosis	0=No 1=_____years	Therapeutic drugs	0=No 1=(_____year____month)	0=No 1=Yes	□__years	□__years	□__years	□__years	□	□
**E16.** **<u>Last year</u>**, you visited a doctor__________times; you were hospitalized for_________times.										
**E17.** **<u>During the last two weeks</u>**, whether your body is sick or not? 0=No (*<u>Go to **E18**</u>*) 1=Yes										□
**E17a.** 1= No treatment, no self-medication or adjuvant therapy 2= Did not see a doctor, but took the drug or took some adjuvant therapy 3= Go to the medical unit for treatment										□

**E18-19 (only married women)**	
**E18.** Did doctor tell you that you had hypertension during your pregnancy? 0=No 1=Yes 9=Unknown □	
**E19.** Did doctor tell you that you had diabetes during your pregnancy? 0=No 1=Yes 9=Unknown □	
**Menstruation and reproductive history (only women)**	
**F1.** How many times do you have been pregnant:_____times. How many births did you have:_____times	
**F2.** How many miscarriages did you have:_____times, of which, the times of abortion:_____times	
**F3.** Have you ever had a breastfeeding experience? 0=No 1=Yes (cumulative_______years)	
**F4.** Have you taken a contraceptive pill or a contraceptive injection?	
0=No (*Go to **F5***) 1=Yes (cumulative_______years)	
**F5.** The age of your first menstrual period is_______years old.	
**F6.** Are you menopausal? 0=No 1=Yes 2= Surgery (resection of the uterus, ovaries) □	
0=No	**F6a.** Are you pregnant now? 0=No 1=Yes 2=Possible □
1=Yes	**F6e.** If it is (surgery), how old are you menopausal (when surgery)? □

**Emotion and pressure** **(Please choose the option that best suits you)**				
**G1.** **Last month**, what do you think about your psychological pressure? □<br>1=None 2=Low 3=Moderate 4=High 5= Extremely high				
**G2.** Here are five questions(**G2a-G2e**), please choose the option that best suits you (over the last two weeks).				
**Cases**	Not at all	Several days	More than half the days	Nearly every day
**G2a.** Little interest or pleasure in doing things	1	2	3	4
**G2b.** Feeling low, depressed, or hopeless	1	2	3	4
**G2c.** Feeling nervous, anxious, or on edge.	1	2	3	4
**G2d.** Not being able to stop or control worrying.	1	2	3	4
**G2e.** Have the idea of " It's better to die " or hurt yourself in some way	1	2	3	4
**Body Functions** **(Please choose the option that best suits you)**				
**G3.** Having problems in walking around： □<br>1=None 2= Slightly 3=Moderately 4=Severely 5= Extremely				
**G4.** Having problems washing or dressing myself： □<br>1=None 2= Slightly 3=Moderately 4=Severely 5= Extremely				
**G5.** Having problems doing usual activities (e.g. work, study, housework): □<br>1=None 2= Slightly 3=Moderately 4=Severely 5= Extremely				
**G6.** Having pain or discomfort： □<br>1=None 2= Slightly 3=Moderately 4=Severely 5= Extremely				
**G7.** Being anxious or depressed： □				

1=None 2= Slightly 3=Moderately 4=Severely 5= Extremely
**G8.** Please assess your today's health condition with a score ranged 0-100: _____<br>(The higher the score, the healthier you are)<br>├──┼──┼──┼──┼──┼──┼──┼──┼──┼──┤<br>0 10 20 30 40 50 60 70 80 90 100
Signature of investigator: __________ End time of this survey: _____h_____mins
Please access the quality of this survey: 1=High 2=Moderate 3=Low □

Physical examination

First **Second**

H1. Height ___________cm ___________cm (Accurate to 0.1 cm)

H2. Waist ___________cm ___________cm (Accurate to 0.1 cm)

H3. Hip ___________cm ___________cm (Accurate to 0.1 cm)

Signature of investigator： _______________

H4. Weight ___________ kg (Accurate to 0.1 kg)

H5. Body fat percentage ___________ % (Accurate to 0.1%)

H6. Basic metabolism ___________ kcal

H7. Visceral fat index___________

Signature of investigator： __________

Left hand / Right hand

H8. The first reading ________ / ________kg (Accurate to 0.1 kg)

H9. The second reading ________ / ________kg (Accurate to 0.1 kg)

H10. The third reading ________ / ________kg (Accurate to 0.1 kg)

H11. Dominant hand 1= Left 2=Right □

Signature of investigator： _____________

H12. Blood pressure & heart rate (to be measured after sitting quietly for five minutes)

The first reading ____________/__________mmHg ____________times/min

The second reading ____________/__________mmHg ____________times/min

The third reading ____________/__________mmHg ____________times/min

Signature of investigator： _____________
